# No Association between Cortical Gyrification or Intrinsic Curvature and Attention-deficit/Hyperactivity Disorder in Adolescents and Young Adults

**DOI:** 10.3389/fnins.2017.00218

**Published:** 2017-04-20

**Authors:** Natalie J. Forde, Lisa Ronan, Marcel P. Zwiers, Aaron F. Alexander-Bloch, Stephen V. Faraone, Jaap Oosterlaan, Dirk J. Heslenfeld, Catharina A. Hartman, Jan K. Buitelaar, Pieter J. Hoekstra

**Affiliations:** ^1^Department of Psychiatry, University of Groningen, University Medical Center GroningenGroningen, Netherlands; ^2^Department of Cognitive Neuroscience, Donders Institute for Brain, Cognition and Behaviour, Radboud University Medical CenterNijmegen, Netherlands; ^3^Brain Mapping Unit, Department of Psychiatry, University of CambridgeCambridge, UK; ^4^Child Psychiatry Branch, National Institute of Mental HealthBethesda, MD, USA; ^5^Departments of Psychiatry and of Neuroscience and Physiology, SUNY Upstate Medical UniversitySyracuse, NY, USA; ^6^Department of Biomedicine, K.G. Jebsen Centre for Research on Neuropsychiatric Disorders, University of BergenBergen, Norway; ^7^Clinical Neuropsychology Section, Department of Clinical, Neuro and Developmental Psychology, Vrije Universiteit AmsterdamAmsterdam, Netherlands; ^8^Department of Experimental and Applied Psychology, Vrije Universiteit AmsterdamAmsterdam, Netherlands; ^9^Karakter Child and Adolescent Psychiatry University CentreNijmegen, Netherlands

**Keywords:** ADHD, intrinsic curvature, biomarker, connectivity, gyrification, development

## Abstract

Magnetic resonance imaging (MRI) studies have highlighted subcortical, cortical, and structural connectivity abnormalities associated with attention-deficit/hyperactivity disorder (ADHD). Gyrification investigations of the cortex have been inconsistent and largely negative, potentially due to a lack of sensitivity of the previously used morphological parameters. The innovative approach of applying intrinsic curvature analysis, which is predictive of gyrification pattern, to the cortical surface applied herein allowed us greater sensitivity to determine whether the structural connectivity abnormalities thus far identified at a centimeter scale also occur at a millimeter scale within the cortical surface. This could help identify neurodevelopmental processes that contribute to ADHD. Structural MRI datasets from the NeuroIMAGE project were used [*n* = 306 ADHD, *n* = 164 controls, and *n* = 148 healthy siblings of individuals with ADHD (age in years, mean(sd); 17.2 (3.4), 16.8 (3.2), and 17.7 (3.8), respectively)]. Reconstructions of the cortical surfaces were computed with FreeSurfer. Intrinsic curvature (taken as a marker of millimeter-scale surface connectivity) and local gyrification index were calculated for each point on the surface (vertex) with Caret and FreeSurfer, respectively. Intrinsic curvature skew and mean local gyrification index were extracted per region; frontal, parietal, temporal, occipital, cingulate, and insula. A generalized additive model was used to compare the trajectory of these measures between groups over age, with sex, scanner site, total surface area of hemisphere, and familiality accounted for. After correcting for sex, scanner site, and total surface area no group differences were found in the developmental trajectory of intrinsic curvature or local gyrification index. Despite the increased sensitivity of intrinsic curvature, compared to gyrification measures, to subtle morphological abnormalities of the cortical surface we found no milimeter-scale connectivity abnormalities associated with ADHD.

## Introduction

Attention-deficit/hyperactivity disorder (ADHD) is a common neurodevelopmental disorder affecting~5% of the school age population (Polanczyk et al., 2007) and characterized by pervasive inattention and/or hyperactivity and impulsivity leading to impairments of functioning (American Psychiatric Association, 2013).

ADHD has been proposed to be a dysconnectivity disorder (Konrad and Eickhoff, 2010) where neural circuits are implicated rather than regions, and there has been a move toward investigating ADHD, and other disorders, in terms of connectivity and integration instead of segregation; where specific regional abnormalities are implicated (Friston, 2011). This shift has come in both functional and structural studies, with recent diffusion magnetic resonance imaging (dMRI) analysis concentrating on network connectivity based on white matter tracts as opposed to the traditional voxel-based or region-of-interest analyses (Cao et al., 2013; Hong et al., 2014). A meta-analysis and contemporary review of the available dMRI data revealed that multiple white matter tracts are affected in ADHD, including the anterior corona radiata, forceps minor, and superior and inferior longitudinal fasciculi (Liston et al., 2011; van Ewijk et al., 2012). These white matter tracts consist of bundles of long-range axonal fibers that connect distant gray matter regions (e.g., cortical to sub-cortical structures, inter-hemispheric connections, or frontal to parietal lobes, etc.). However, it is not established whether these long-range white matter connectivity differences are echoed in short-range connections within the cortex. Interestingly, despite the cortex generally being associated with cell bodies rather than connections 95% of connections in the brain are found in the cortex in the form of short-range connections (Braitenberg and Schüz, 1998). Within this study we therefore ask; are these long range abnormalities echoed in the short range connections within the cortex?

Evidence from previous studies does suggest abnormalities of the cortex in those with ADHD. To date many routine markers, such as cortical thickness and volume, have been used to report structural changes in the cortex of individuals with ADHD; however, these results are non-specific in relation to connectivity or the underlying cytoarchitecture of the cortex and sometimes inconsistent (Shaw et al., 2007, 2013; Wolosin et al., 2009; Nakao et al., 2011; Frodl and Skokauskas, 2012; Schweren et al., 2015). Studies into cortical thickness and surface area measures suggest that cortical development has a delayed developmental trajectory in ADHD, with both surface area and cortical thickness reaching their peak later in individuals with ADHD (Shaw et al., 2007, 2012).

**Figure 1:**
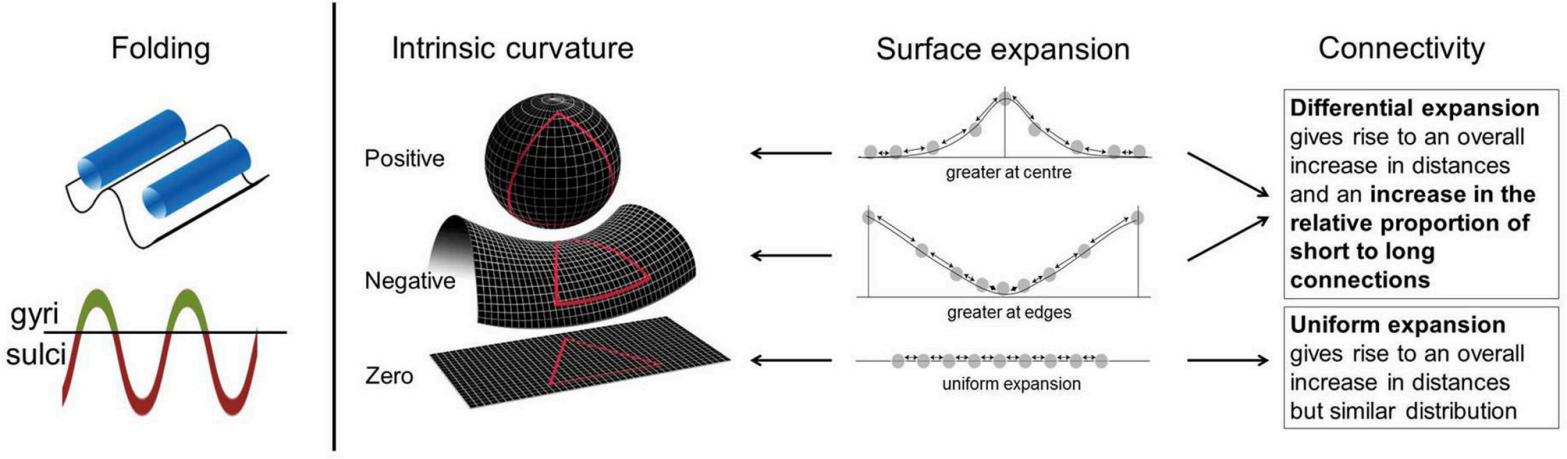
**Intrinsic curvature and folding**. Folding of the cortical surface, indexed by gyrification measures, relates to extrinsic curvature i.e., how the surface is bent in 3-dimensional space. No intrinsic curvature of the surface is required for folding. Surface expansion is depicted schematically in the above figure with points on a line, equal distribution of points before expansion is assumed. Arrows between points can be interpreted as connections. Differential expansion of the cortex results in either positive or negative intrinsic curvature values depending on whether expansion is faster at the center or edges of the surface. Uniform expansion (zero intrinsic curvature) results in an overall increase in distances between points but no change in the proportion of long to short connections. Differential expansion (positive or negative intrinsic curvature) also results in an increase in the overall distance between points but more importantly it also increases the relative proportion of short to long connections. Figure adapted in part from Ronan et al. (2011, 2012).

It is, however, not clear whether previous cortical findings also relate to connectivity abnormalities within the cortex. To address this we used cortical intrinsic curvature. This is a morphological measure of the intrinsic deformation of the surface and as such may be interpreted in terms of the underlying connectivity of the cortex (Ronan et al., 2011, 2012). This is in contrast to extrinsic measures, such as gyrification, which are related to the embedding of the cortex in three-dimensional space rather than the engrained curvature of the surface (Figure 1). These distinct metrics of surface shape are measured at the millimeter-scale (intrinsic curvature) and centimeter-scale respectively (gyrification). The important distinction between these parameters is the nature of the shape they capture. Intrinsic curvature is measure of deformation—i.e., the stretching or compression of the surface, while gyrification (indexed here by the local gyrification index) is a marker of folding. Importantly folding does not deform the surface itself (i.e., distances along the surface remain the same; think of a line drawn on a piece of paper—the length of the line is not changed whether the paper is folded or not). On the other hand deformation (as captured by intrinsic curvature) changes distances along the surface (again, the length of a line on a surface is changed if the surface is stretched or compressed). With the application of intrinsic curvature analysis of the cortical surface we are able to investigate the millimeter-scale connectivity of axonal processes within the gray matter of the cortex. Differential expansion is the process whereby the surface does not expand uniformly but instead has various rates of expansion across the cortex during development resulting in a fluctuating pattern of positive and negative intrinsic curvatures. This differential expansion underlies intrinsic curvature and also results in a greater range of inter-neuronal distances which skews the length distribution toward having a higher proportion of shorter connections, from which more efficient connectivity may be inferred (Figure 1, Ronan et al., 2011, 2012). We could therefore make use of the relationship of differential expansion to both intrinsic curvature and connectivity to use one (intrinsic curvature) as a quantifiable measure of the other (connectivity). Describing intrinsic curvature abnormalities associated with ADHD would therefore support the dysconnectivity theory of ADHD by implicating the involvement of short range connections. Intrinsic curvature could then potentially be used clinically as a biological marker of ADHD. Null findings in relation to ADHD would suggest that connectivity abnormalities are constrained to long range connections within the white matter. Either way, this would aid in furthering our understanding of ADHD and its etiology.

Intrinsic curvature has previously been used and shown to be sensitive to cortical differences related to connectivity in patients affected by schizophrenia compared to healthy controls (Ronan et al., 2012) and in a study of healthy participants with various combinations of the *brain derived neurotrophic factor* (*BDNF)* val66met polymorphism (Forde et al., 2014). Furthermore, the related measure of *wiring cost* was found altered in a group of adults with autism spectrum disorder (ASD; Ecker et al., 2013). ASD has also been associated with gyrification and long range connectivity abnormalities (Anagnostou and Taylor, 2011; Schaer et al., 2013). ADHD and ASD share many characteristics as neurodevelopmental disorders and, at least partly, their heritability (Rommelse et al., 2010, 2011), adding weight to our hypothesis that there may be short range connectivity abnormalities in the cortex of individuals with ADHD.

Intrinsic curvature is distinct from, though related to, the overall degree of gyrification (Ronan et al., 2012). While gyrification abnormalities in the left medial temporal region (Mous et al., 2014) and folding abnormalities in the right frontal lobe (Wolosin et al., 2009) have been reported in children with ADHD in two small studies, such abnormalities were not seen in a larger study of gyrification by Shaw et al. (2012). It has been demonstrated that the move in scale-sensitivity using intrinsic curvature, from centimeter to millimeter, increases the power to detect subtle shape differences in the cortex indicative of abnormal neurodevelopment (Ronan et al., 2012). We therefore investigated both local gyrification index and intrinsic curvature in the current study with the assumption that the largely negative previous gyrification studies of ADHD (Shaw et al., 2012) may have been obfuscated by the scale of morphological parameters employed. We thus hypothesized that an investigation of gyrification within the current study would similarly show no group differences while intrinsic curvature would detect subtle morphological alterations indicative of short range dysconnectivity in ADHD.

ADHD is a highly heritable (~80%), genetically complex and heterogeneous disorder (Faraone et al., 2005). Endophenotypes, biologically based phenotypes, hold much promise as less genetically complex markers underlying psychiatric conditions thereby allowing the pathophysiology of conditions to be elucidated (Gottesman and Gould, 2003). As endophenotypes can be thought of as markers of the genetic liability of a disorder they should appear in those with a shared genetic heritage irrespective of diagnosis, for instance the unaffected relatives of an affected individual (Gottesman and Gould, 2003). We therefore took this opportunity to additionally explore the potential of intrinsic curvature as an endophenotypic marker of ADHD by including healthy siblings of those with ADHD, along with the individuals with ADHD and healthy controls in our study design.

## Methodology

### Participants

This study was undertaken under the remit of the NeuroIMAGE study, for details see von Rhein et al. (2014) and the study website (www.neuroimage.nl). Briefly the NeuroIMAGE study is the follow up, within the Netherlands, of the International Multicenter ADHD Genetics study (IMAGE; Müller et al., 2011a,b). Initially families who had an individual with ADHD-combined type and healthy control families were recruited to the IMAGE study; all participants were Caucasian, aged 6–18 years and had an IQ ≥ 70. Exclusion criteria were a diagnosis of autism, epilepsy and brain, or genetic disorders. Within the ADHD families, individuals with psychiatric diagnoses (other than ADHD) were excluded except for oppositional defiant disorder (ODD), conduct disorder (CD), and pervasive developmental disorder not otherwise specified (PDD-NOS). One or more subjects with ADHD and one or more healthy sibling of those with ADHD from the same family were included. Similarly, multiple healthy subjects were included from healthy control families to balance the familial effect across groups. An extensive battery of diagnostic and neuropsychological tests as well as genetic data were acquired for all participants. From the Dutch sites (Vrije Universiteit [VU] in Amsterdam and Radboud UMC in Nijmegen), all initial participants were invited to participate in the follow up (mean follow up 5.9 years), namely NeuroIMAGE, where neuroimaging data were acquired in addition to behavioral data similar to the initial visit. Note that all ADHD participants were required to still meet criteria for an ADHD diagnosis at time of scanning, therefore those who remitted were omitted from this analysis.

There were 618 full datasets from 374 different families available for the current analysis. Of these there were 306 participants with ADHD (mean [*SD*] age 17.2 [3.4] years), 148 healthy siblings of an individual with ADHD (mean [*SD*] age 17.7 [3.8] years), and 164 healthy controls (mean [*SD*] age 16.8 [3.2] years), see Table 1 for full demographic details.

**Table 1:** **Group Demographics**.

	**ADHD**		**Siblings**	**Control**	**Test statistic**	***p*-value**
*n*	306		148	164	–	–
Age in years, mean (*SD*)	17.2 (3.4)		17.7 (3.8)	16.8 (3.2)	K–W χ^2^ = 4.71	0.095
Sex, m/f	208/98		62/86	87/77	χ^2^ = 29.85	<0.001^***^
IQ, mean (*SD*)	97.0 (15.2)		102.8 (14.3)	105.6 (13.5)	K–W χ^2^ = 36.71	<0.001^***^
Scanner site Ams/Nij	150/156		78/70	105/59	χ^2^ = 9.78	0.008^*^
Handedness r/l/a	269/33/3		124/18/3	146/13/4	χ^2^ = 3.31	0.51
ADHD Symptom count, *n (SD*)	13.2 (3.0)		1.2 (1.9)	0.8 (1.7)	K–W χ^2^ = 484.61	<0.001^***^
^a^Stimulant use (never/previous/current)	41/112/147		134/8/1	145/0/0		
Comorbidities	No	Yes	–	–	–	–
	147	159	–	–	–	–
ODD &/or CD only		116	–	–	–	–
^b^Multiple or other		43	–	–	–	–

SD, standard deviation, m/f–male/female, r/l/a–right/left/ambidextrous, ODD, oppositional defiant disorder, CD, conduct disorder, K–W, Kruskal-Wallis test, χ^2^–chi squared test. Scanner site relates to the number of data sets that were acquired at each of the two sites in the study; Ams, VU Amsterdam and Nij, Radboud UMC, Nijmegen.

a Medication data were not available for all participants (missing for: 6, 5, and 19 participants from the ADHD, sibling and healthy control groups, respectively).

b This included 22 additional cases of ODD &/or CD along with 10 cases of tic disorders and 33 cases of mood disorders.

* p < 0.05,

*** *p < 0.001*.

At the time of follow-up, all participants in the study were similarly assessed using a combination of a semi-structured diagnostic interview conducted by trained professionals (Dutch translation of the Schedule for Affective Disorders and Schizophrenia for School-Age Children—Present and Lifetime Version K-SADS; Kaufman et al., 1997) and combination of Conners' ADHD questionnaires, these rating were collected of children's functioning off medication. Each child was assessed with a parent-rated questionnaire (Conners' Parent Rating Scale—Revised: Long version CPRS-R:L; Conners et al., 1998a) combined with either a teacher-rating (Conners' Teacher Rating Scale—Revised: Long version CTRS-R:L; Conners et al., 1998b) or a self-report (Conners' Adult ADHD Rating Scales—Self-Report:Long Version CAARS-S:L; Conners et al., 1999).

A diagnostic algorithm was applied to combine symptom counts from the K-SADS and Conners' questionnaires. ADHD diagnosis was given to participants with a combined total symptom count of ≥ 6 (≥ 5 for participants ≥ 18 years) of hyperactive/impulsive and/or inattentive behavior, provided they also: (a) met the DSM-IV criteria for pervasiveness, impact of the disorder and onset-age before 12, and (b) scored T ≥ 63 on at least one of the Conners' questionnaires (parent, teacher, or self-rating). Healthy control participants were required to score T < 63 on both of the Conners' questionnaires, and have ≤ 3 (≤ 2 for participants ≥ 18 years). The K-SADS was additionally used to assess ODD, CD, and presence of tics. Full details can be found in von Rhein et al. (2014).

### Structural MRI acquisition

Two T1-weighted MPRAGE scans were acquired for each participant at one of the two test sites (Amsterdam and Nijmegen). Similar 1.5 Tesla MRI scanners were employed (Siemens SONATA and Siemens AVANTO; Siemens, Erlangen, Germany), using identical head coils (8-channel Phase Array Head Coil). Images were acquired with a sagittal, 3-dimentional GRAPPA parallel imaging sequence with the following parameters: TE = 2.95 ms, TR = 2730 ms, TI = 1000 ms, flip angle = 7°, voxel dimension = 1 × 1 × 1 mm and acquisition time 6.21 min.

### Quality assessment

Image quality was assessed manually by two independent judges. The better quality scan was selected for each participant and those with poor quality scans were omitted (*n* = 14; already excluded from the demographic descriptions).

### Surface reconstruction

The cortical surfaces were reconstructed using FreeSurfer v5.3 (Dale et al., 1999; Fischl et al., 1999a,b; Fischl and Dale, 2000), a programme specifically designed for cortical reconstruction and volumetric segmentation (Dale et al., 1999; Fischl et al., 1999a,b; Fischl and Dale, 2000). The raw images were fed into the programme where the voxels were subsampled to voxels of 1 mm^3^, normalized for intensity, RF-bias field inhomogeneities were removed and the images skull stripped. The gray-white border was then identified followed by the hemispheres being separated, tessellated and deformed resulting in a smooth representation of the pial and white matter surfaces.

### Intrinsic curvature

Intrinsic curvature was calculated per vertex of each participants FreeSurfer reconstruction using Caret software (v5.65, http://brainvis.wustl.edu/wiki/index.php/Caret:About). This process has been detailed previously (Forde et al., 2014; Ronan et al., 2014). The Caret-generated files of intrinsic curvature were imported to MatLab where they underwent filtering to remove outlier curvature values that were not feasible given the resolution of cortical reconstruction (Ronan et al., 2012, 2014). Absolute values of the remaining per vertex intrinsic curvature measures were calculated. Per region the skew of the curvature distribution was then calculated (Ronan et al., 2012, 2014). These regions (frontal, parietal, occipital, temporal, cingulate, and insula) were generated by combining labels from the Desikan-Killiany Atlas (Desikan et al., 2006) which is supplied with the FreeSurfer package. Cortical intrinsic curvature has a distribution highly skewed toward zero intrinsic curvature (Pienaar et al., 2008; Ronan et al., 2011, 2012), therefore the less skewed the distribution, the greater the degree of intrinsic curvature and differential expansion.

### Local gyrification index

Gyrification index (GI) is the ratio of the amount of cortical surface exposed as opposed to buried within sulcal folds. A large GI indicates a highly folded surface. Local gyrification index (lGI) quantifies GI at each vertex on the surface and is computed in a 3D fashion by using a region of interest around each vertex within the FreeSurfer software (Schaer et al., 2008). Mean local gyrification index was then extracted per region.

### Statistical analysis

R statistics programme was used for all statistical analysis and graph generation. Continuous group demographics; age, IQ, and symptom count were investigated for normality of distribution (Shapiro-Wilks test) and homogeneity of variance (Bartlett's test). Following this, if the assumptions of normality and homogeneity were met, group differences were investigated with an one-way analysis of variance (ANOVA) or, if one or more of the assumptions were violated, with the non-parametric equivalent, the Kruskal-Wallis test.

The non-linear trajectories of intrinsic curvature skew and local gyrification index over age, based on our cross-sectional data, were modeled per group using a generalized additive mixed-effect (GAM) model approach (Wood, 2006) allowing us to compare the developmental trajectories for the different groups. Applying a GAM model allowed the non-linear modeling of the relationship between age and intrinsic curvature skew with greater flexibility than the standard polynomial form of the growth curve. This method has previously been effectively applied in neuroimaging data (Alexander-Bloch et al., 2014). Briefly, penalized spline mixed-effect models were used to fit the developmental trajectories for each group in each region. This was done using the gamm4 (Wood and Scheipl, 2014) and mcgv (Wood, 2011) packages in R statistics with sex, scanner site, and surface area included as possible confounders. Total cortical surface area was included to control for brain size as both intrinsic curvature and gyrification develop as a function of surface expansion. The non-independence of family members was accounted for by including family as a random factor. IQ, stimulant use, comorbidity, and symptom severity were added to the model to investigate their effect. Due to the nature of additive models group-by-age and group-by-sex interactions could not be appropriately modeled within the GAM model and were instead modeled using a linear mixed effect model (LME) with similar settings to the GAM model. As there were no hemisphere-by-group interactions, measures were collapsed from left and right to give an average intrinsic curvature skew or average local gyrification index per region which was used for analyses. To account for multiple comparisons (two measures each tested in six regions) the alpha level was adjusted to 0.004 for all tests.

### Sensitivity analysis

Due to the possible confounds of having groups ill matched for sex and scanner site a sensitivity analysis was undertaken. Individuals were carefully matched on sex, scanner site, and age which resulted in a subset of participants (*n* = 66 per group, see Table 2). Furthermore, all participants with ADHD and a comorbid condition (ODD, CD, tic disorder etc.) were excluded. The above statistical methods were then reapplied to this subset.

**Table 2:** **Demographics from matched groups**.

	**ADHD**	**Siblings**	**Control**	**Test stat**	***p*-value**
*n*	66	66	66	–	–
Age in years, mean (*SD*)	16.97 (2.67)	17.03 (2.73)	17.07 (2.67)	0.02	0.98
Age in years, range	11.3–22.0	11.3–22.2	11.5–22.5	–	–
Sex, m/f	38/28	38/28	38/28	–	–
Scanner site, Ams/Nij	29/37	29/37	29/37	–	–
IQ, mean (*SD*)	99.45 (14.1)	99.6 (14.1)	103.6 (11.5)	2.06	0.13
Handedness r/l/a	59/7/0	55/7/1	60/4/2		
Symptom count, *n* (*SD*)	13.08 (2.94)	1.39 (2.03)	0.79 (1.77)		

*SD, standard deviation, m/f, male/female, r/l/a, right/left/ambidextrous*.

## Results

### Demographics

Groups did not differ significantly with respect to age. Groups did differ with respect to the proportion of males to females, the distribution of subjects across the two scanner sites and IQ. Therefore, these measures were included in further analysis (Table 1). Also approximately half of the ADHD group had one or more comorbid conditions. A total of 138 participants with ADHD had comorbid ODD and/or CD, including 130 subjects with ODD and 46 subjects with a diagnosis of CD. Ten participants with ADHD also presented with tics. Thirty-three were also diagnosed with a mood disorder. There were 147 participants with ADHD and no comorbidities. Those with comorbidities were excluded from the sensitivity analysis to remove the possibility that these had an effect on findings.

### Intrinsic curvature

**Figure 2:**
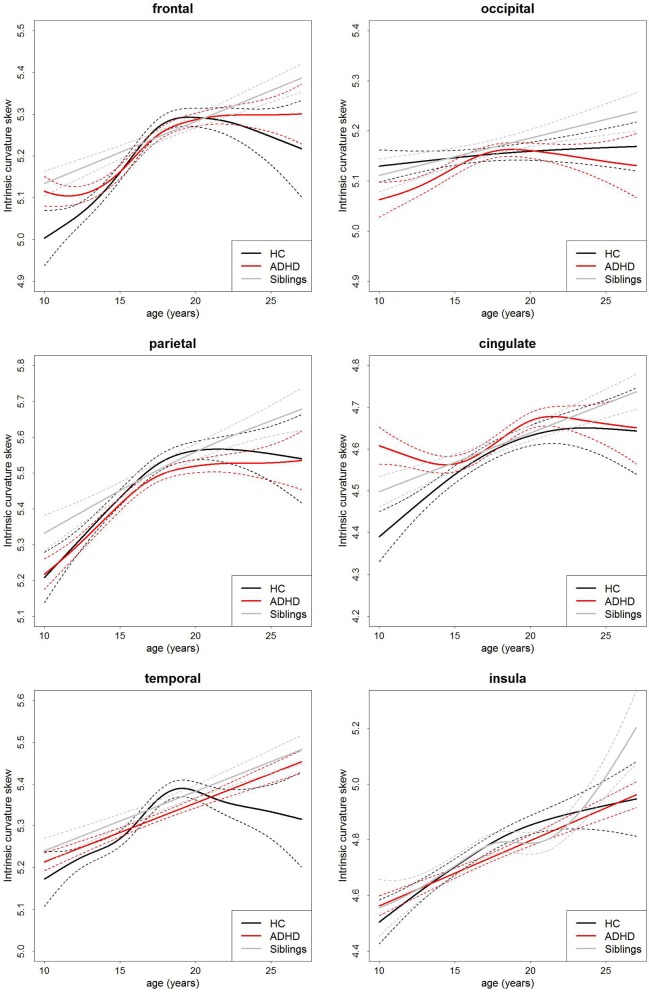
**Age-curves of intrinsic curvature skew per group for each region**. Differences between groups were not significant. Caution must be taken when viewing these graphs as a very small proportion of the participants were under the age of 12 or over 23 years of age thus the apparent differences at these ages are driven by a few individuals only. Broken lines represent the standard error for each group. HC, healthy control (black lines), ADHD, Attention-deficit/hyperactivity disorder (red lines), Siblings, healthy siblings of ADHD participant (gray lines).

There was no main effect of group on intrinsic curvature (Table 3, Figure 2). Indicating no difference in the degree of differential expansion, and therefore the underlying cytoarchitechture and connectivity of the cortex, between individuals with ADHD, their siblings and controls. There was a very strong main effect of age in all regions (Table 3). There was also a main effect of sex in the frontal region (intrinsic curvature skew higher in females; *t* = 4.11, *p* = 4.57 × 10^−5^) while in the temporal and cingulate regions total surface area was also significant (*t* = −5.78, *p* = 1.19 × 10^−8^ and *t* = 3.47, *p* = 0.0006, respectively).

**Table 3:** **Results**.

	**Intrinsic curvature**				**Local gyrification index**			
**Region**	**Group F**	**Group *p***	**Age F**	**Age *p***	**Group F**	**Group *p***	**Age F**	**Age *p***
Frontal	0.41	0.66	128.75	9.6 × 10^−25^^***^	0.63	0.53	326.90	1.3 × 10^−60^^***^
Parietal	1.96	0.14	108.31	3.0 × 10^−21^^***^	0.41	0.67	407.01	2.2 × 10^−74^^***^
Temporal	0.74	0.48	63.95	5.6 × 10^−15^^***^	0.26	0.77	192.43	5.0 × 10^−36^^***^
Occipital	2.61	0.07	10.58	0.001^*^	0.71	0.49	116.53	5.6 × 10^−22^^***^
Cingulate	2.94	0.05	40.31	4.4 × 10^−8^^***^	0.66	0.52	92.26	1.3 × 10^−20^^***^
Insula	0.56	0.57	60.20	3.3 × 10^−14^^***^	1.83	0.16	129.14	3.3 × 10^−24^^***^

Test statistics and p-values are reported for the main effects of group and age on intrinsic curvature and local gyrification index in each region for the full sample (n = 618). Adjusted p = 0.004.

* p < 0.004,

*** *p < 8 × 10^−5^*.

### Local gyrification index

**Figure 3:**
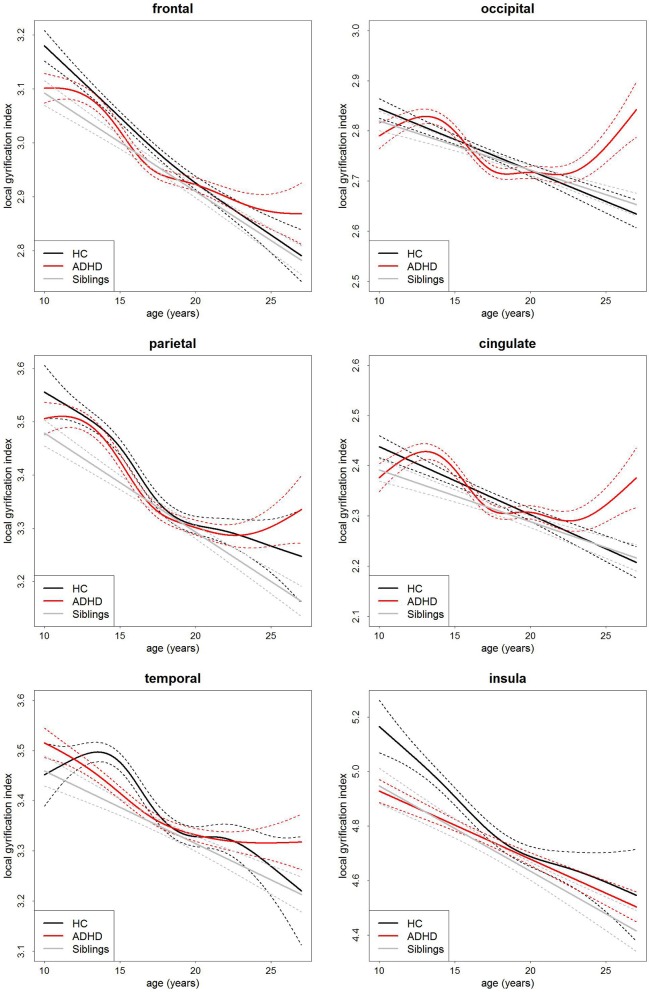
**Age-curves of local gyrification index per group for each region**. Differences between groups were not significant. Caution must be taken when viewing these graphs as a very small proportion of the participants were under the age of 12 or over 23 years of age thus the apparent differences at these ages are driven by a few individuals only. Broken lines represent the standard error for each group. HC, healthy control (black lines), ADHD, Attention-deficit/hyperactivity disorder (red lines), Siblings, healthy siblings of ADHD participant (gray lines).

Similarly, there was no main effect of group on local gyrification (Table 3, Figure 3). This implies there is no differences in the degree of cortical folding between participants with ADHD, their siblings and controls. There was a very strong main effect of age (Table 3) and total surface area (frontal: *t* = 14.12, *p* = 2.37 × 10^−39^, parietal: *t* = 17.11, *p* = 8.25 × 10^−54^, temporal: *t* = 19.31, *p* = 4.17 × 10^−65^, occipital: *t* = 14.50, *p* = 4.41 × 10^−41^, cingulate: *t* = 13.13, *p* = 7.90 × 10^−35^, and insula: *t* = 14.07, *p* = 4.10 × 10^−39^) in all regions.

Neither IQ, symptom severity, comorbidity nor stimulant status had an effect on the intrinsic curvature or local gyrification models. There were also no significant group-by-age or group-by-sex interactions in either the intrinsic curvature or local gyrification analyses as modeled with a LME model. Finally, the sensitivity analysis to ensure that neither the covariates (sex and scanner site) nor comorbidities were confounding our study revealed no group differences between the carefully matched groups (Table 4). Furthermore, data analyzed per test site and per sex showed similar findings in each case (see Supplementary Tables 1–4).

**Table 4:** **Matched results**.

	**Intrinsic curvature**				**Local gyrification index**			
**Region**	**Group F**	**Group *p***	**Age F**	**Age *p***	**Group F**	**Group *p***	**Age F**	**Age *p***
Frontal	0.15	0.86	36.55	6.8 × 10^−9^^***^	0.32	0.72	90.98	1.2 × 10^−16^^***^
Parietal	1.04	0.36	37.91	3.8 × 10^−9^^***^	0.29	0.75	103.33	1.5 × 10^−18^^***^
Temporal	1.16	0.32	29.50	1.6 × 10^−7^^***^	0.06	0.94	47.12	7.1 × 10^−11^^***^
Occipital	0.52	0.59	4.28	0.04	0.90	0.41	15.88	9.5 × 10^−5^^**^
Cingulate	2.25	0.11	20.99	8.1 × 10^−6^^***^	1.32	0.27	15.35	1.2 × 10^−4^^**^
Insula	2.23	0.11	6.98	0.009	0.13	0.88	37.70	4.6 × 10^−8^^***^

Test statistics and p-values are reported for the main effects of group and age on intrinsic curvature and local gyrification index in each region for the matched sample (n = 198). Adjusted p = 0.004. ^*^p < 0.004,

** p < 8 × 10^−4^,

*** *p < 8 × 10^−5^*.

### Tics

Only 10 members of the ADHD group were also seen to have tics, this low number (3.3%) may relate to the older age of participants (average 17.22 years) and method of recruiting (specifically recruiting families affected by ADHD) and meant statistical analysis between those with and without tics was deemed futile due to the lack of power.

## Discussion

We applied measures of cortical intrinsic curvature and local gyrification to investigate differences in cortical brain development, related to cortical connectivity, between people with ADHD, their healthy siblings and unrelated healthy controls. We found no difference between the groups with respect to either intrinsic curvature or local gyrification index within any of the regions investigated.

These negative findings indicate that developmental abnormalities previously found in the cortex of those with ADHD (Shaw et al., 2007, 2012) are not due to underlying differences in differential expansion. ADHD has been associated with cortical developmental delay of measures such as cortical thickness and surface area (Shaw et al., 2007, 2012) and cross sectional abnormalities of cortical volume and thickness (Filipek et al., 1997; Makris et al., 2007; Wolosin et al., 2009; Almeida et al., 2010; Proal et al., 2011; Almeida Montes et al., 2012; Frodl and Skokauskas, 2012), this includes cortical thickness deficits bilaterally in the medial temporal cortex that have previously been reported in this study cohort (Schweren et al., 2015). This large study of gyrification is in keeping with a previous longitudinal study that showed no maturational differences in gyrification between individuals with ADHD compared to healthy controls (Shaw et al., 2012). However, two smaller studies have previously reported differences between those with ADHD and controls; in gyrification of the left medial temporal region (Mous et al., 2014) and folding index globally and in the right frontal lobe (Wolosin et al., 2009). Inconsistency in findings may relate to various methods having been employed. We proposed that intrinsic curvature analysis may have been more sensitive than gyrification measures to detect cortical differences between groups if present, however, our results concur with the previous finding of Shaw et al. (2012) in that we found no diagnostic difference in intrinsic curvature, which is predictive of gyrification pattern (Ronan et al., 2014).

In contrast to our hypothesis we can infer from this that there are no short range cortico-cortico connectivity differences within the gray matter of the cortex between those with ADHD, their siblings or healthy controls. Previous reports have found evidence of white matter connectivity abnormalities in ADHD when long range connections between distinct gray matter regions were analyzed. Our findings suggest that these changes do not similarly occur at a smaller within gray matter scale but are constrained to the white matter. Furthermore, this finding helps differentiate ADHD from ASD which has been associated with cortical connectivity abnormalities in adults (Ecker et al., 2013) and schizophrenia where the cortical connectivity differences seen (Ronan et al., 2012) are proposed to relate to the abnormal cytoarchitechture present in schizophrenia (Selemon et al., 1995, 1998). As well as cortical connectivity differences, abnormalities in white matter tracts have been shown in schizophrenia (Ellison-Wright and Bullmore, 2009; Ellison-Wright et al., 2014) and ASD (Barnea-Goraly et al., 2004; Alexander et al., 2007). While larger scale connectivity differences also occur in ADHD (Konrad and Eickhoff, 2010) from this study we can infer that, unlike in schizophrenia and ASD, there are no short range connectivity abnormalities in the cortical gray matter of ADHD patients. This implies that despite a shared heritability between ASD and ADHD (Rommelse et al., 2010) there are, at least partially, different abnormal developmental mechanisms at play in the respective conditions.

Given our null findings of differences between groups the use of either IC or LGI alone do not seem to be sensitive endophenotypic markers for ADHD. However, despite this, considering the high heritability of cortical indices (Thompson et al., 2001; Panizzon et al., 2009; Rogers et al., 2010) the inclusion of these measures along with various other biological and cognitive indices in more complex data driven approaches may aid in identifying biomarkers and endophenotypes for ADHD.

Intrinsic curvature holds much potential as a sensitive marker of cortical connectivity and abnormal cortical development. However, it has not yet been widely used and how the measure changes over the lifetime in healthy participants needs further quantification. Although our study had substantial numbers of participants (*n* = 618) we lacked the power to detect differences in the early adolescent and early adulthood stages of development. This is due to our age range being normally distributed about our mean, resulting in robust findings through mid to late adolescents but reduced power in early adolescence and adulthood. Finally, interactions between group and age were modeled using a standard linear mixed-effects model, which showed no significant interactions, instead of the GAM model. This was due to the nature of additive models which by definition do not allow interactions. However, there remains the possibility that there may well be an interaction between group and age but that this is not discernible with a linear model.

In conclusion, we found there are no short range connectivity differences within the cortical gray matter, as inferred from intrinsic curvature measures, between participants with ADHD, their unaffected siblings and healthy controls.

## Ethics statement

The protocol was approved by the Commissie Mensgebonden Onderzoek (CMO) Regio Arnhem-Nijmegen and the medical ethical committee of the VU University Medical Center. For children between 12 and 18 both parents and children gave written informed consent. For participants below 12 parents gave written informed consent.

## Author contributions

NF analyzed these data and wrote the manuscript. LR, MZ, and AA significantly contributed to the processing and/or analysis of data. SF, JO, DH, CH, JB, and PH were all involved with the conception and funding of the NeuroIMAGE project. CH and LR further acted as statistical experts. While JB and PH supervised the study and critically evaluated the manuscript.

### Conflict of interest statement

JB has been in the past 3 years a consultant to/member of advisory board of/and/or speaker for Janssen Cilag BV, Eli Lilly, Shire, Medice, Lundbeck, Roche, and Servier. He is not an employee of any of these companies, and not a stock shareholder of any of these companies. He has no other financial or material support, including expert testimony, patents, royalties. The other authors declare that the research was conducted in the absence of any commercial or financial relationships that could be construed as a potential conflict of interest.

## Abbreviations

ADHD: attention-deficit/hyperactivity disorder.
