# Supplementary material for: No Association between Cortical Gyrification or Intrinsic Curvature and Attention-deficit/Hyperactivity Disorder in Adolescents and Young Adults
Source: Front Neurosci. 2017 Apr 20;11:218. doi: 10.3389/fnins.2017.00218 (PMC5397412; doi:10.3389/fnins.2017.00218)
Supplement: Supplementary file 1 [file Table1.docx]

Supplementary information

|  | **Intrinsic Curvature** | | | | **Local Gyrification Index** | | | |
| --- | --- | --- | --- | --- | --- | --- | --- | --- |
| **Region** | **Group F** | **Group *p*** | **Age F** | **Age *p*** | **Group F** | **Group *p*** | **Age F** | **Age *p*** |
| **Frontal** | 0.26 | 0.77 | 68.70 | 1.8 ×10^-13^ | 2.33 | 0.10 | 162.92 | 2.9 ×10^-30^ |
| **Parietal** | 0.73 | 0.48 | 45.77 | 7.8 ×10^-10^ | 0.15 | 0.86 | 233.98 | 7.6 ×10^-43^ |
| **Temporal** | 1.24 | 0.29 | 47.49 | 2.3 ×10^-11^ | 0.98 | 0.38 | 96.14 | 2.3 ×10^-19^ |
| **Occipital** | 0.98 | 0.38 | 5.36 | 0.02 | 0.27 | 0.76 | 77.82 | 3.8 ×10^-15^ |
| **Cingulate** | 1.86 | 0.16 | 23.88 | 6.3 ×10^-5^ | 0.13 | 0.88 | 39.94 | 7.5 ×10^-10^ |
| **Insula** | 1.15 | 0.32 | 39.58 | 8.8 ×10^-10^ | 4.06 | 0.02 | 51.95 | 3.1 ×10^-12^ |

Supplementary Table 1: Test statistics and *p*-values for the main effects of group and age on intrinsic curvature and local gyrification index in each region for male participants only (n=357).

|  | **Intrinsic Curvature** | | | | **Local Gyrification Index** | | | |
| --- | --- | --- | --- | --- | --- | --- | --- | --- |
| **Region** | **Group F** | **Group *p*** | **Age F** | **Age *p*** | **Group F** | **Group *p*** | **Age F** | **Age *p*** |
| **Frontal** | 0.12 | 0.89 | 47.64 | 1.5 ×10^-9^ | 1.58 | 0.21 | 120.94 | 2.0 ×10^-22^ |
| **Parietal** | 1.83 | 0.16 | 55.78 | 5.0 ×10^-11^ | 0.30 | 0.74 | 160.56 | 2.3 ×10^-28^ |
| **Temporal** | 0.18 | 0.84 | 29.12 | 3.4 ×10^-6^ | 3.29 | 0.04 | 77.64 | 8.7 ×10^-17^ |
| **Occipital** | 1.86 | 0.16 | 3.36 | 0.07 | 1.48 | 0.23 | 37.59 | 3.1 ×10^-9^ |
| **Cingulate** | 1.68 | 0.19 | 14.46 | 1.8 x10^-4^ | 1.80 | 0.17 | 41.94 | 4.3 ×10^-10^ |
| **Insula** | 0.13 | 0.88 | 23.50 | 2.1 ×10^-6^ | 0.29 | 0.75 | 57.43 | 4.8 ×10^-13^ |

Supplementary Table 2: Test statistics and *p*-values for the main effects of group and age on intrinsic curvature and local gyrification index in each region for female participants only (n=261).

|  | **Intrinsic Curvature** | | | | **Local Gyrification Index** | | | |
| --- | --- | --- | --- | --- | --- | --- | --- | --- |
| **Region** | **Group F** | **Group *p*** | **Age F** | **Age *p*** | **Group F** | **Group *p*** | **Age F** | **Age *p*** |
| **Frontal** | 0.09 | 0.91 | 52.34 | 2.6 ×10^-10^ | 0.08 | 0.92 | 134.44 | 2.7 ×10^-24^ |
| **Parietal** | 1.10 | 0.34 | 37.52 | 6.3 ×10^-8^ | 0.05 | 0.95 | 145.23 | 1.2 ×10^-27^ |
| **Temporal** | 5.54 | 0.00 | 23.06 | 1.5 ×10^-5^ | 0.45 | 0.64 | 74.04 | 3.1 ×10^-16^ |
| **Occipital** | 1.78 | 0.17 | 6.98 | 0.009 | 0.03 | 0.97 | 46.45 | 5.1 ×10^-11^ |
| **Cingulate** | 1.82 | 0.16 | 8.70 | 0.003 | 1.01 | 0.37 | 43.41 | 2.0 ×10^-10^ |
| **Insula** | 1.58 | 0.21 | 22.91 | 2.7 ×10^-6^ | 0.25 | 0.78 | 53.26 | 3.7 ×10^-11^ |

Supplementary Table 3: Test statistics and *p*-values for the main effects of group and age on intrinsic curvature and local gyrification index in each region for participants from the Nijmegen site only (n=285).

|  | **Intrinsic Curvature** | | | | **Local Gyrification Index** | | | |
| --- | --- | --- | --- | --- | --- | --- | --- | --- |
| **Region** | **Group F** | **Group *p*** | **Age F** | **Age *p*** | **Group F** | **Group *p*** | **Age F** | **Age *p*** |
| **Frontal** | 0.13 | 0.88 | 62.61 | 2.9 ×10^-14^ | 0.97 | 0.38 | 188.76 | 1.5 ×10^-33^ |
| **Parietal** | 0.90 | 0.41 | 64.76 | 1.3 ×10^-12^ | 0.53 | 0.59 | 267.65 | 2.9 ×10^-47^ |
| **Temporal** | 1.04 | 0.36 | 32.15 | 3.0 ×10^-8^ | 0.42 | 0.66 | 119.72 | 1.2 ×10^-21^ |
| **Occipital** | 0.34 | 0.71 | 2.81 | 0.09 | 1.68 | 0.19 | 60.69 | 6.7 ×10^-12^ |
| **Cingulate** | 0.54 | 0.58 | 30.39 | 7.1 ×10^-6^ | 0.15 | 0.86 | 45.18 | 7.3 ×10^-11^ |
| **Insula** | 0.15 | 0.86 | 34.77 | 8.9 ×10^-9^ | 1.70 | 0.18 | 70.81 | 2.6 ×10^-13^ |

Supplementary Table 4: Test statistics and *p*-values for the main effects of group and age on intrinsic curvature and local gyrification index in each region for participants from the Amsterdam site only (n=333).
